# miR-18a-5p promotes phenotypic transformation of airway smooth muscle cells by targeting SPRY1 to activate the RAS-MAPK pathway

**DOI:** 10.3389/fgene.2026.1760431

**Published:** 2026-03-19

**Authors:** Lijuan Hu, Lei Li

**Affiliations:** Department of Emergency Medicine, Shanxi Bethune Hospital, Shanxi Academy of Medical Sciences, Third Hospital of Shanxi Medical University, Tongji Shanxi Hospital, Taiyuan, China

**Keywords:** airway, asthma, bronchial, miR-18a-5p, phenotypic, remodeling, transformation

## Abstract

**Objective:**

This study investigates the role of miR-18a-5p in the phenotypic transformation of airway smooth muscle cells (ASMCs) and its underlying mechanism in asthma-related airway remodeling.

**Methods:**

Expression of miR-18a-5p in sputum from asthma patients was assessed by RT-qPCR. An *in vitro* model was established by stimulating ASMCs with TGF-β1. The responsiveness of miR-18a-5p expression to asthma-related mitogenic stimulation was also evaluated. ASMC proliferation and migration were evaluated following miR-18a-5p overexpression or inhibition using CCK-8 and Transwell assays. Western blot was used to detect migration-associated proteins, phenotypic markers and activation of the RAS-MAPK pathway. The regulatory relationship between miR-18a-5p and SPRY1 was validated by dual-luciferase assay, and SPRY1 knockdown was performed to explore its functional role.

**Results:**

miR-18a-5p was significantly upregulated in asthma patients. Overexpression of miR-18a-5p promoted ASMC proliferation and migration, accompanied by upregulation of migration-related proteins (Integrin β1,p-FAK/FAK,p-Paxillin/Paxillin,MMP9), increased synthetic phenotype markers (α-SMA, OPN, Collagen I/III), and reduced contractile marker (Calponin). It also activated the RAS-MAPK pathway. SPRY1 was confirmed as a direct target of miR-18a-5p. Knockdown of SPRY1 reversed the effects of miR-18a-5p inhibition, confirming its role in mediating ASMC phenotypic changes.

**Conclusion:**

miR-18a-5p promotes ASMC phenotypic switching and airway remodeling in asthma by targeting SPRY1 and activating the RAS-MAPK signaling pathway. These findings suggest miR-18a-5p as a potential therapeutic target for asthma.

## Introduction

Bronchial asthma is a chronic inflammatory airway disease that can affect individuals of all age groups, characterized by persistent local inflammation, reversible airway obstruction, airway hyperresponsiveness, and airway remodeling (Chung et al., 2022). In recent years, the incidence and prevalence of asthma have shown a continuous upward trend globally, particularly in economically underdeveloped regions such as Asia and Latin America, due to factors including increased environmental pollution and allergen exposure (Asher et al., 2020). According to the latest epidemiological investigations, approximately 358 million individuals worldwide are affected by asthma (Stern et al., 2020). This disease severely impacts patients' physical development and quality of life, while also imposing a considerable healthcare burden on families and society. Current therapeutic regimens primarily include inhaled corticosteroids, long-acting β2-adrenergic receptor agonists, and leukotriene receptor antagonists (Garner et al., 2022; Agache et al., 2021). Although these medications are partially effective in symptom control and inflammation suppression, several limitations persist. Some patients develop drug tolerance or dependency with prolonged use, leading to reduced efficacy and adverse effects such as growth retardation and osteoporosis (Wu et al., 2022; Palumbo et al., 2020). Moreover, these treatments mainly target the inflammatory response and bronchoconstriction and are unable to fundamentally delay or reverse airway remodeling, thereby failing to prevent disease progression and chronicity (Castil et al., 2017). Therefore, elucidating the mechanisms underlying asthma pathogenesis and identifying new therapeutic targets is critical for the development of safer and more effective treatment strategies that can fundamentally halt disease progression and alleviate the societal burden.

Airway remodeling is a hallmark pathological feature during the chronic course of bronchial asthma and is primarily manifested as epithelial cell injury, thickening of the subepithelial basement membrane, mucus gland hyperplasia, neovascularization, and significant increases in the number and size of airway smooth muscle cells (ASMCs) (Savin et al., 2023). Chronic inflammatory stimuli and recurrent airway responses contribute to the development of airway remodeling, ultimately resulting in airway narrowing, airflow limitation, and decreased pulmonary function, all of which adversely affect disease control and patient quality of life (Huang and Qiu, 2022). ASMCs, located in the submucosa, are among the principal structural cells in the airway wall and are responsible for maintaining airway tone and respiratory function under physiological conditions. However, due to their inherent plasticity, ASMCs readily respond aberrantly to inflammatory stimuli under pathological conditions, undergoing structural and functional changes that make them central to the pathogenesis of airway remodeling (Camoretti-Mercado and Lockey, 2021). In asthma, ASMCs undergo a phenotypic switch from a dormant, contractile phenotype to a synthetic phenotype. Synthetic ASMCs exhibit cellular hypertrophy, accelerated proliferation, enhanced migration, and excessive secretion of cytokines, thereby contributing to airway wall thickening, increased airway tension, and exaggerated hyperresponsiveness (Rosethorne and Charlton, 2018). Thus, targeting the regulatory mechanisms of ASMC phenotypic transformation may represent a promising strategy to mitigate airway remodeling and improve asthma outcomes.

MicroRNAs (miRNAs) are a class of highly conserved, endogenous, non-coding small RNAs approximately 22 nucleotides in length that regulate gene expression primarily through complementary binding to the 3′untranslated regions (3′UTRs) of target mRNAs. miRNAs are widely involved in physiological and pathological processes such as cell proliferation, differentiation, apoptosis, migration, and tissue development (Ho et al., 2022). In recent years, accumulating evidence has revealed a significant role for miRNAs in the pathogenesis of asthma. For instance, Panganiban et al. found that plasma levels of miR-206, miR-125b, miR-16, miR-133b, and miR-299 were abnormally expressed in asthma patients, distinguishing them from healthy individuals (Panganiban et al., 2016). Similarly, He et al. demonstrated significant differences in serum levels of miR-106a-5p, miR-18a-5p, miR-44-3p, and miR-375 between asthmatic children and healthy controls (He et al., 2022). Among the various asthma-associated miRNAs, miR-18a-5p has emerged as a molecule of particular interest. Studies have shown that miR-18a-5p plays a critical role in regulating immune and inflammatory responses. For example, Egeland et al. reported a strong correlation between miR-18a-5p expression and immune cell infiltration in breast cancer tissues, suggesting its involvement in shaping the inflammatory tumor microenvironment (Egeland et al., 2020). Furthermore, research by Trenkmann et al. revealed that miR-18a-5p can establish a sustained positive feedback loop by upregulating matrix-degrading enzymes, inflammatory mediators, and chemokines, thereby aggravating rheumatoid arthritis pathology (Trenkmann et al., 2013). These findings support the pro-inflammatory nature of miR-18a-5p. However, despite the chronic inflammatory nature of asthma and observed dysregulation of miR-18a-5p in asthma patients, its precise mechanistic role in asthma remains inadequately understood.

Based on the above, we hypothesize that miR-18a-5p may participate in airway remodeling by modulating ASMC phenotypic transformation. To test this hypothesis, we established an *in vitro* ASMC model induced by transforming growth factor-β1 (TGF-β1) to comprehensively evaluate the regulatory effects of miR-18a-5p. Furthermore, by employing miR-18a-5p overexpression and inhibition along with dual-luciferase reporter assays, we aimed to confirm its targeting interaction with SPRY1 and elucidate the downstream signaling pathways involved. This study will enhance our understanding of the role of miR-18a-5p in asthma-related airway remodeling and provide a theoretical basis for the development of novel therapeutic strategies.

## Materials and methods

### Participants and sputum sample collection

This study enrolled 10 patients diagnosed with asthma and 10 healthy volunteers. Asthma patients were recruited from the Department of Respiratory Medicine (outpatient or inpatient) of Shanxi Bethune Hospital between November 2024 and February 2025, and diagnosed with bronchial asthma based on the Global Initiative for Asthma (GINA) guidelines (Rajvanshi et al., 2024). Only newly diagnosed, treatment-naïve patients in a stable state (not during acute exacerbation) were included to minimize heterogeneity. The following exclusion criteria were applied: (1) history of smoking; (2) diagnosis of chronic pulmonary diseases other than asthma within the past year (e.g., chronic obstructive pulmonary disease, bronchiectasis); (3) severe chronic conditions such as heart failure, chronic kidney disease, or liver disease; and (4) concurrent viral infections or immune system disorders. Healthy controls were recruited from individuals undergoing routine physical examinations at the hospital’s Health Examination Center during the same period. They were age- and gender-matched with the asthma patients, and had no history of asthma, allergic rhinitis, or other allergic diseases, no history of smoking, and no abnormalities on physical or pulmonary function examination.

Sputum samples were collected in the early morning. Prior to collection, all participants rinsed their mouths with sterile water or saline for 10 min to minimize contamination by saliva or nasal secretions. Induced sputum collection was performed using hypertonic saline nebulization. Briefly, participants inhaled aerosolized hypertonic saline (3%–5%) via an ultrasonic nebulizer under the supervision of trained clinical personnel to induce deep coughing and sputum expectoration. Sputum plugs (>3 mL) were collected and processed within 2 h of collection.

Following previously reported protocols (Fu et al., 2017; Yang et al., 2021), each sample was transferred to a 50 mL centrifuge tube, the sputum portion was weighed, and the volume of cell wash solution and DTT was adjusted proportionally (4 volumes of wash solution and 0.1 volume of DTT per 1 volume sputum, following established protocols. The mixture was then shaken on a rocking platform until complete liquefaction. The mixture was centrifuged (2000 rpm, 4 °C, 10 min), the supernatant was discarded, and the cell pellet was collected for subsequent analysis. To ensure sputum sample quality, visible saliva was carefully removed prior to processing. Samples with excessive squamous epithelial cell contamination (>50%) were excluded. Cell viability was assessed using trypan blue exclusion, and only samples with a viability greater than 70% were included for downstream analyses.

This study was approved by the Ethics Committee of Shanxi Bethune Hospital (Approval No. YXLL-2025-012).

### Cell culture

Primary airway smooth muscle cells (ASMCs) derived from mice were obtained from the American Type Culture Collection (ATCC, Manassas, VA, United States) as primary cells and used as the *in vitro* model in this study. Because these cells are primary mouse ASMCs rather than an immortalized cell line, no ATCC catalog number or Cellosaurus RRID is available. The cells were derived from mouse tracheal airway smooth muscle tissue (species: *Mus musculus*; tissue of origin: airway smooth muscle) Mouse-derived ASMCs were selected for mechanistic experiments because they provide a well-established and reproducible *in vitro* system for investigating signaling mechanisms involved in airway remodeling. The cells were cultured in high-glucose Dulbecco’s Modified Eagle’s Medium (DMEM; Sigma-Aldrich, United States) supplemented with 10% fetal bovine serum (FBS) and 1% penicillin/streptomycin solution. Cultures were maintained in a humidified incubator at 37 °C with 5% CO_2_. Cell authentication was performed by the supplier prior to distribution, and the cells have not been reported to be misidentified or cross-contaminated. All experiments were conducted using early-passage primary cells. In addition, the cells were routinely tested for *mycoplasma* contamination and confirmed to be mycoplasma-free prior to use.

### Cell intervention, transfection, and grouping

Solid recombinant human TGF-β1 protein was purchased from MedChemExpress (United States). The protein was initially dissolved in sterile water to prepare a stock solution and subsequently diluted with sterile phosphate-buffered saline (PBS) to various working concentrations for inducing asthma-related phenotypes in ASMCs.

When the cells reached 70%–80% confluence, they were treated with TGF-β1 at final concentrations of 0, 1, 3, 5, or 10 ng/mL for 24 h, based on concentrations reported in previous studies (Li et al., 2015; Chen et al., 2020). RNA was then extracted, and miR-18a-5p expression levels were quantified by real-time quantitative PCR (RT-qPCR). Expression of miR-18a-5p was found to increase in a concentration-dependent manner, reaching its peak at 10 ng/mL TGF-β1; this concentration was therefore selected for subsequent experiments. For downstream functional and mechanistic assays, ASMCs were stimulated with TGF-β1 at 10 ng/mL for 48 h. This stimulation duration was selected based on previous studies in which 48 h of TGF-β1 treatment is commonly used to induce phenotypic and functional changes in ASMCs, including enhanced proliferation and migration, while maintaining acceptable cell viability. (Okazaki et al., 2025). Therefore, this time point was considered appropriate for subsequent analyses.

In addition, to evaluate whether miR-18a-5p expression is responsive to another key mitogenic stimulus involved in airway remodeling, ASMCs were treated with recombinant platelet-derived growth factor-BB (PDGF-BB) at final concentrations of 5, 10, 20, or 40 ng/mL for 24 h. Untreated ASMCs served as the control group.

MiR-18a-5p mimics, inhibitors, their respective negative controls (mi-NC and in-NC), siRNA designed to knock down SPRY1 expression (si-SPRY1), and its negative control (si-NC) were all synthesized and validated by GenePharma (Shanghai, China). Transfections were performed using Lipofectamine 3000 (Invitrogen, United States) according to the manufacturer’s instructions. Following transfection, cells were cultured for 48 h prior to sample collection.

Subsequently, ASMCs were assigned to ten experimental groups: (1) Control group: untreated ASMCs cultured for 48 h; (2) TGF-β1 group: ASMCs stimulated with 10 ng/mL TGF-β1 for 48 h; (3) TGF-β1+in-NC group: ASMCs transfected with negative control inhibitor followed by 10 ng/mL TGF-β1 stimulation for 48 h; (4) TGF-β1+in-miR group: ASMCs transfected with miR-18a-5p inhibitor followed by TGF-β1 stimulation for 48 h; (5) TGF-β1+mi-NC group: ASMCs transfected with negative control mimics followed by TGF-β1 stimulation for 48 h; (6) TGF-β1+mi-miR group: ASMCs transfected with miR-18a-5p mimics followed by TGF-β1 stimulation for 48 h; (7) si-NC group: ASMCs transfected with negative control siRNA for 48 h; (8) si-SPRY1 group: ASMCs transfected with SPRY1 siRNA for 48 h; (9) TGF-β1+si-SPRY1 group: ASMCs transfected with si-SPRY1 followed by TGF-β1 stimulation for 48 h; (10) TGF-β1+in-miR + si-SPRY1 group: ASMCs co-transfected with miR-18a-5p inhibitor and si-SPRY1, then treated with TGF-β1 for 48 h. Transfection efficiency and knockdown efficacy were quantitatively validated. The expression levels of miR-18a-5p following transfection with miR-18a-5p mimics or inhibitors were assessed by RT-qPCR. The efficiency of SPRY1 knockdown by si-SPRY1 was confirmed by RT-qPCR analysis at both the mRNA and protein levels.

### RT-qPCR analysis

Total RNA was extracted from ASMCs and sputum samples using TRIzol reagent (Invitrogen, United States). RNA concentration and purity were measured using a NanoDrop 2000 spectrophotometer (Thermo Fisher Scientific, United States). Subsequently, reverse transcription was performed using the PrimeScript RT Reagent Kit (Takara, Japan) for SPRY1 and the miRNA First-strand cDNA Synthesis Kit (Takara, Japan) for miR-18a-5p. Quantitative RT-qPCR was then conducted on an Applied Biosystems 7500 Real-Time PCR System (Applied Biosystems, United States) with SYBR Premix Ex Taq II (Takara, Japan) to construct the reaction system.

The expression of miR-18a-5p was normalized to U6 small nuclear RNA, and SPRY1 expression was normalized to GAPDH. Relative expression levels were calculated using the 2^-ΔΔCt^ method. Each sample was analyzed in triplicate, and the average value was used as the final result. Primer sequences used in PCR are listed in Table 1.

**TABLE 1:** RT-qPCR primers.

RNA	Sequences (5′ to 3′)
*SPRY1*	5′- GCGTGCTTTGCAGAGTGATT -3’ (forward) 5′- CACGGCCGAAATGCCTAATG -3’ (reverse)
mir-18a-5p	5′- CGCGTAAGGTGCATCTAGTGC -3’ (forward) 5′- AGTGCAGGGTCCGAGGTATT -3’ (reverse)
*GAPDH*	5′- TGGATTTGGACGCATTGGTC -3’ (forward) 5′- TTTGCACTGGTACGTGTTGAT-3’ (reverse)
*U6*	5′- CAGGTCTCCAAGACGACATAGA -3’ (forward) 5′- CGCCTTTTCGATTCATGTACTGC -3’ (reverse)

### ASMC proliferation assay

In this study, the proliferation of ASMCs was evaluated using the Cell Counting Kit-8 (CCK-8; Dojindo, Japan). Briefly, ASMCs subjected to different treatments were seeded into 96-well plates at a density of 3 × 10^4^ cells/well. Each well was supplemented with 10 μL of CCK-8 solution and incubated under standard conditions for 72 h. After incubation, optical density (OD) was measured at 450 nm using a multifunctional microplate reader (Thermo Fisher Scientific, United States) to assess the number of viable cells, which reflected cell proliferation capacity. Each experimental condition was performed in triplicate, and the mean value was recorded as the final result.

### Transwell assay

The migratory capacity of ASMCs was assessed using a Transwell assay. After treatment, cells from each group were cultured in serum-free high-glucose DMEM for 24 h to synchronize their state. Subsequently, cells were resuspended in serum-free medium and gently seeded into the upper chambers of 24-well Transwell inserts (8.0 μm pore size; Corning, United States). The lower chambers were filled with high-glucose DMEM supplemented with 15% fetal bovine serum (FBS) as a chemoattractant. Cells were incubated for 48 h.

After incubation, the medium in the upper chambers was carefully removed, and the chambers were washed with PBS. Non-migrated cells on the upper surface were gently wiped off with a cotton swab. The membranes were then fixed in 4% paraformaldehyde for 30 min and stained with 0.1% crystal violet for 1 h. Migrated cells were observed and counted under an optical microscope (Olympus, Japan) in five randomly selected fields per well. Each experiment was repeated in triplicate, and the average value was used for subsequent analysis.

### Immunofluorescence staining

ASMCs from each treatment group were seeded into 6-well plates containing sterile round glass coverslips. After cell adhesion, cells were gently washed twice with PBS to remove residual culture medium. The cells were then fixed with 4% paraformaldehyde at room temperature for 30 min, permeabilized with 0.1% Triton X-100 (Sigma-Aldrich, United States) for 10 min, and blocked with 3% bovine serum albumin (BSA; Solarbio, China) for 30 min at room temperature.

Following blocking, the cells were incubated overnight at 4 °C in the dark with primary antibodies diluted in blocking buffer: anti-α-SMA (1:200, 14-9760-82, Invitrogen, United States) and anti-Collagen I (1:200, PA1-26204, Invitrogen, United States). The next day, the coverslips were washed with PBS and incubated with corresponding fluorophore-conjugated secondary antibodies for 1 h at room temperature in the dark. After washing again with PBS, the cells were mounted using a DAPI-containing antifade mounting medium (Abcam, United Kingdom).

Fluorescent images were acquired using a fluorescence microscope (Olympus, Japan). For each group, five randomly selected fields were photographed. ImageJ software (NIH, United States) was used to perform semi-quantitative fluorescence intensity analysis to evaluate the expression levels of α-SMA and Collagen I in ASMCs. Each experiment was conducted in triplicate, and the average value was used as the final result.

### Western blot analysis

Total protein was extracted from ASMCs of each group after treatment using RIPA lysis buffer (Thermo Fisher Scientific, United States). Protein concentrations were determined with a bicinchoninic acid (BCA) protein assay kit (Abcam, United Kingdom). Equal amounts of protein samples were separated by 10% SDS-PAGE and transferred to polyvinylidene difluoride (PVDF) membranes (Abcam, United Kingdom). Membranes were then blocked with 5% non-fat milk at room temperature for 1 h.

Subsequently, membranes were incubated overnight at 4 °C with the following primary antibodies: anti-Integrin β1 (1:1,000, ab179471, United Kingdom); FAK (1:1,000, ab40794, United Kingdom); p-FAK (1:1,000, ab81298, United Kingdom); Paxillin (1:1,000, ab32115, United Kingdom); p-Paxillin (1:1,000, ab194738, United Kingdom); MMP9 (1:1,000, ab283575, United Kingdom); anti-OPN (1:1,000, PA5-34579, Invitrogen, United States); anti-α-SMA (1:1,000, 14-9760-82, Invitrogen, United States); anti-Calponin (1:1,000, MA5-11620, Invitrogen, United States); anti-Collagen I (1:2,000, PA1-26204, Invitrogen, United States); anti-Collagen III (1:2,000, MA5-42628, Invitrogen, United States); anti-KRAS (1:1,000, 415700, Invitrogen, United States); anti-MEK1/2 (1:1,000, MA5-15162, Invitrogen, United States); anti-p-MEK1/2 (1:1,000, MA5-15016, Invitrogen, United States); anti-ERK1/2 (1:1,000, 13-6200, Invitrogen, United States); anti-p-ERK1/2 (1:1,000, 14-9109-82, Invitrogen, United States); anti-SPRY1 (1:1,000, PA5-18289, Invitrogen, United States); and anti-α-tubulin (1:4,000, PA5-19489, Invitrogen, United States).

After washing, the membranes were incubated with horseradish peroxidase (HRP)-conjugated secondary antibodies at room temperature for 2 h. Immunoreactive bands were visualized using the Odyssey infrared imaging system (LiCor, United States). Band intensities were quantified using ImageJ software (NIH, United States), and target protein expression levels were normalized to α-tubulin.

### Dual-luciferase reporter assay

A dual-luciferase reporter assay was performed to verify the putative targeting relationship between miR-18a-5p and SPRY1. First, potential binding sites of miR-18a-5p within the 3′UTR of SPRY1 were predicted using the ENCORI tool in the StarBase online database (https://rnasysu.com/encori/). The wild-type (WT) SPRY1 3′UTR sequence containing the predicted binding site and a mutated (MUT) version with altered binding sequences were synthesized and inserted into the pGL3 luciferase reporter vector (VectorBuilder, China), resulting in the pGL3-SPRY1-WT and pGL3-SPRY1-MUT constructs.

HEK293T cells were co-transfected with either miR-18a-5p mimics or negative control (mi-NC) along with the respective reporter plasmids. After 48 h, luciferase activity was measured using the Dual Luciferase Reporter Gene Assay Kit (Beyotime, China). Firefly luciferase activity was used for reporter detection, and Renilla luciferase served as an internal control. The normalized Firefly/Renilla ratio was calculated to evaluate the interaction between miR-18a-5p and SPRY1. Each experiment was performed in triplicate, and the average value was used for statistical analysis.

### Statistical analysis

All statistical analyses were performed using IBM SPSS Statistics software (version 26.0). Data distribution was assessed for normality prior to statistical analysis. Comparisons between two groups were performed using an unpaired Student’s t-test. Comparisons among multiple groups were conducted using one-way ANOVA followed by Tukey’s *post hoc* test to correct for multiple comparisons. The correlation between miR-18a-5p and SPRY1 expression levels in sputum samples was evaluated using Pearson’s correlation analysis. For analyses involving clinical sputum specimens, each group consisted of 10 independent samples. Each *in vitro* experiment was independently repeated three times. A P value <0.05 was considered statistically significant.

Given the exploratory nature of this study, no formal *a priori* sample size or statistical power calculation was performed. The sample size was determined based on feasibility and the availability of well-characterized asthma patients and healthy controls, with the aim of providing preliminary clinical evidence to support subsequent mechanistic investigations.

## Results

### Upregulation of miR-18a-5p in asthma patients and its role in promoting TGF-β1-induced ASMC proliferation and migration

**FIGURE 1:**
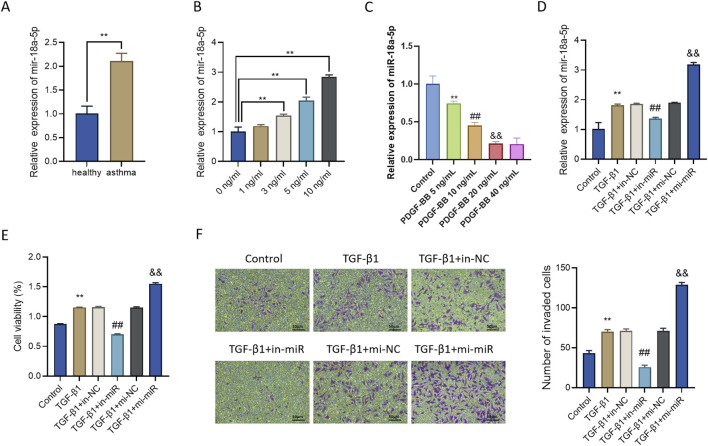
Upregulation of miR-18a-5p in asthma and its role in promoting ASMC proliferation and migration upon TGF-β1 stimulation. **(A)** RT-qPCR revealed that miR-18a-5p levels were significantly higher in sputum from asthma patients compared with healthy controls (n = 10). **(B)** TGF-β1 stimulation induced a dose-dependent increase in miR-18a-5p expression in ASMCs (n = 3). **(C)** PDGF-BB stimulation significantly altered miR-18a-5p expression in ASMCs in a concentration-dependent manner (n = 3). **(D)** miR-18a-5p was effectively overexpressed or silenced in ASMCs following transfection with mimics or inhibitors (n = 3). **(E)** CCK-8 assay showed altered cell viability under different miR-18a-5p expression levels (n = 3). **(F)** Transwell assay demonstrated that miR-18a-5p regulates the migratory capacity of ASMCs (n = 3). Data are presented as mean ± SD. **p < 0.01 vs. healthy, 0 ng/mL, or Control group; ##p < 0.01 vs. TGF-β1+in-NC or PDGF-BB 5 ng/mL group; &&p < 0.01 vs. TGF-β1+mi-NC or PDGF-BB 10 ng/mL group.

To investigate whether miR-18a-5p expression differs between asthma patients and healthy individuals, we first performed RT-qPCR analysis. The results revealed that the expression level of miR-18a-5p in sputum samples from asthma patients was significantly higher than that in healthy controls (Figure 1A), suggesting a potential pro-pathogenic role of miR-18a-5p in asthma.

To determine the optimal concentration of TGF-β1 for stimulating ASMCs, cells were treated with 0, 1, 3, 5, and 10 ng/mL TGF-β1, and the expression of miR-18a-5p was assessed. As shown in Figure 1B, miR-18a-5p expression increased in a dose-dependent manner with TGF-β1 concentration. Specifically, expression levels were significantly elevated in the 3, 5, and 10 ng/mL groups compared with the 0 ng/mL control group, with the highest expression observed at 10 ng/mL. Thus, 10 ng/mL TGF-β1 was selected for subsequent experiments.

Given that platelet-derived growth factor-BB (PDGF-BB) is another potent mitogen implicated in airway remodeling, we further examined the effect of PDGF-BB on miR-18a-5p expression. RT-qPCR analysis demonstrated that PDGF-BB stimulation significantly altered miR-18a-5p expression in ASMCs in a concentration-dependent manner (Figure 1C), indicating that miR-18a-5p is responsive to multiple pro-remodeling stimuli.

To modulate miR-18a-5p expression, we transfected ASMCs with miR-18a-5p mimics or inhibitors. RT-qPCR results confirmed that miR-18a-5p expression was significantly increased in the TGF-β1+mi-miR group compared with the TGF-β1+mi-NC group, and significantly decreased in the TGF-β1+in-miR group compared with the TGF-β1+in-NC group (Figure 1D), indicating successful transfection and effective manipulation of miR-18a-5p levels.

Since uncontrolled proliferation and migration are key biological features of ASMC phenotypic transformation (Chetty and Nielsen, 2021), we next examined changes in proliferation and migration under different treatments. CCK-8 assays demonstrated that the proliferative activity of ASMCs in the TGF-β1+mi-miR group was significantly higher than in the TGF-β1+mi-NC group, whereas the TGF-β1+in-miR group showed reduced cell viability compared with the TGF-β1+in-NC group (Figure 1E). Similarly, Transwell assays showed that miR-18a-5p overexpression enhanced, while its inhibition attenuated, the migratory capacity of ASMCs compared with their respective negative controls (Figure 1F).

**FIGURE 2:**
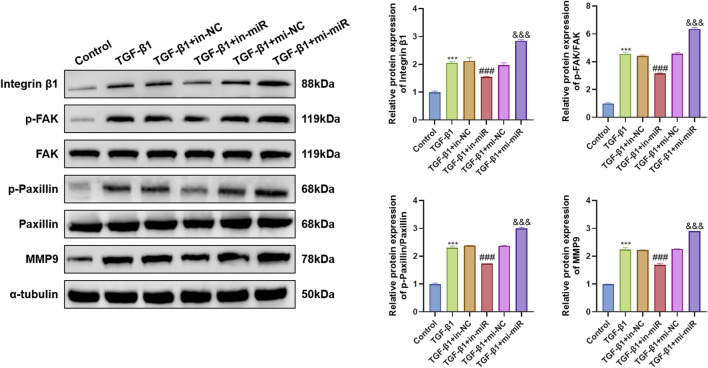
miR-18a-5p regulates migration-associated proteins in TGF-β1-stimulated ASMCs. Representative Western blot images and quantitative analysis showed the expression levels of Integrin β1, p-FAK, total FAK, p-Paxillin, total Paxillin, and MMP9. α-Tubulin was used as a loading control. Data are presented as mean ± SD (n = 3). ***p < 0.001 vs. Control group; ###p < 0.001 vs. TGF-β1+in-NC group; &&&p < 0.001 vs. TGF-β1+mi-NC group.

To further elucidate the molecular basis underlying the pro-migratory effect of miR-18a-5p in TGF-β1-stimulated ASMCs, we examined the expression of migration-associated proteins. Western blot analysis showed that TGF-β1 treatment increased the expression of Integrin β1, p-FAK, p-Paxillin, and MMP9. Overexpression of miR-18a-5p further enhanced these changes, whereas inhibition of miR-18a-5p markedly attenuated the expression of these migration-related proteins (Figure 2). These results provide mechanistic support for the enhanced migratory capacity of ASMCs induced by miR-18a-5p under TGF-β1 stimulation.

Collectively, these findings indicate that miR-18a-5p promotes TGF-β1-induced proliferation and migration of ASMCs.

### miR-18a-5p regulates the expression of structural proteins in TGF-β1-induced ASMCs

Changes in the expression of structural proteins represent one of the most direct indicators of phenotypic transformation in ASMCs (Yu et al., 2022). To evaluate the regulatory effect of miR-18a-5p on TGF-β1-induced phenotypic alterations, we assessed the expression levels of several phenotype-associated markers.

**FIGURE 3:**
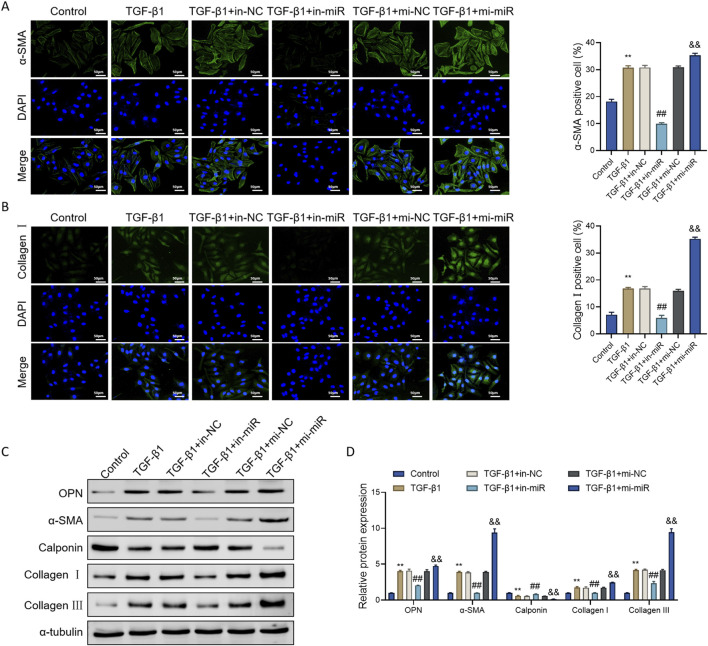
miR-18a-5p modulates the expression of structural proteins in TGF-β1-induced ASMCs. **(A,B)** Immunofluorescence staining of α-SMA and Collagen I in ASMCs subjected to different treatments. **(C)** Representative Western blot images of OPN, α-SMA, Calponin, Collagen I, and Collagen III in ASMCs following different interventions. **(D)** Quantitative analysis of protein expression levels based on Western blot results. Data are presented as mean ± SD (n = 3). **p < 0.01 vs. Control group; ##p < 0.01 vs. TGF-β1+in-NC group; &&p < 0.01 vs. TGF-β1+mi-NC group.

Immunofluorescence analysis revealed that, compared with the Control group, TGF-β1 stimulation markedly increased the expression of α-smooth muscle actin (α-SMA) and Collagen I in ASMCs. Transfection with miR-18a-5p inhibitor (TGF-β1+in-miR group) significantly suppressed the expression of both proteins, whereas overexpression of miR-18a-5p (TGF-β1+mi-miR group) further enhanced their expression (Figures 3A,B).

Western blot analysis further confirmed that TGF-β1 stimulation upregulated the protein levels of osteopontin (OPN), α-SMA, Collagen I, and Collagen III, while downregulating Calponin expression. Inhibition of miR-18a-5p reversed these changes, resulting in reduced levels of OPN, α-SMA, Collagen I, and Collagen III, and an increase in Calponin expression. Conversely, miR-18a-5p overexpression mimicked the effects of TGF-β1, reinforcing the induction of the synthetic phenotype (Figures 3C,D).

Since OPN, α-SMA, Collagen I, and Collagen III are considered markers of the synthetic phenotype, while Calponin is characteristic of the contractile (dormant) phenotype (Yu et al., 2022), these findings suggest that TGF-β1 induces phenotypic switching of ASMCs from a contractile to a synthetic state, and that miR-18a-5p significantly promotes this transition.

### miR-18a-5p enhances RAS-MAPK pathway activation in TGF-β1-induced ASMCs

Aberrant activation of the RAS-MAPK signaling pathway is recognized as a pivotal contributor to airway remodeling in asthma (Freund-Michel and Frossard, 2008). To explore whether miR-18a-5p exerts its function through this pathway, we assessed the expression of key RAS-MAPK-related proteins.

**FIGURE 4:**
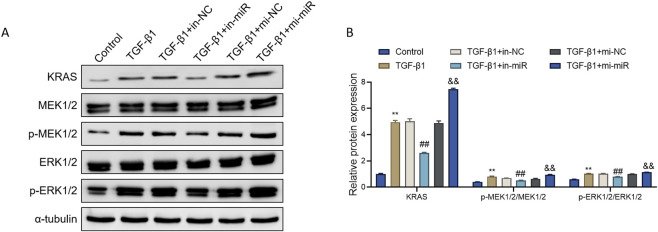
miR-18a-5p enhances RAS-MAPK pathway activation in TGF-β1-induced ASMCs. **(A)** Representative Western blot images of KRAS, MEK1/2, p-MEK1/2, ERK1/2, and p-ERK1/2 protein levels in ASMCs subjected to different treatments. **(B)** Quantitative analysis of protein expression based on Western blot results. Data are presented as mean ± SD (n = 3). **p < 0.01 vs. Control group; ##p < 0.01 vs. TGF-β1+in-NC group; &&p < 0.01 vs. TGF-β1+mi-NC group.

Western blot analysis revealed that TGF-β1 stimulation significantly upregulated the expression of KRAS, phosphorylated MEK1/2 (p-MEK1/2), and phosphorylated ERK1/2 (p-ERK1/2), compared with the Control group. The total levels of MEK1/2 and ERK1/2 also showed moderate increases, and the ratios of p-MEK1/2 to MEK1/2 and p-ERK1/2 to ERK1/2 were markedly elevated, indicating activation of the RAS-MAPK pathway. Inhibition of miR-18a-5p (TGF-β1+in-miR group) led to a notable reduction in the expression and phosphorylation of these proteins, suggesting suppression of pathway activity. Conversely, overexpression of miR-18a-5p (TGF-β1+mi-miR group) further enhanced the expression of KRAS, p-MEK1/2, and p-ERK1/2 compared with the TGF-β1+mi-NC group, indicating a potentiated activation of the signaling cascade (Figures 4A,B).

These results indicate that TGF-β1 can robustly activate the RAS-MAPK signaling pathway in ASMCs, and that miR-18a-5p further amplifies this activation.

### miR-18a-5p directly targets and negatively regulates SPRY1

We next investigated the potential target genes regulated by miR-18a-5p.

**FIGURE 5:**
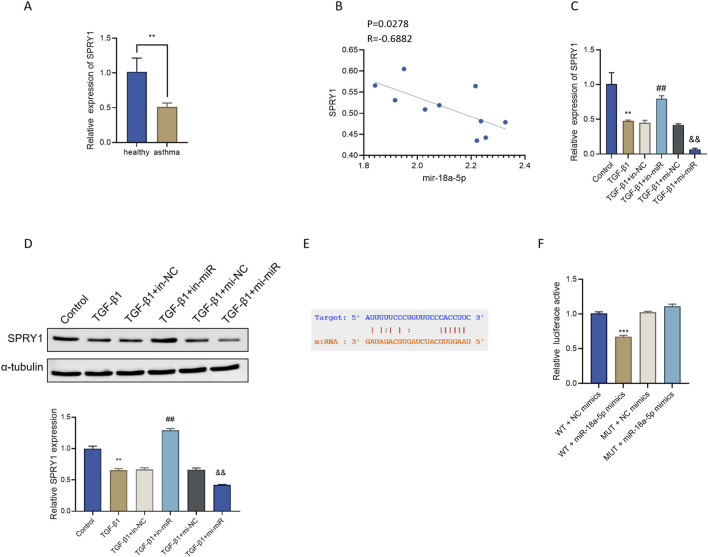
miR-18a-5p targets and negatively regulates SPRY1 **(A)** RT-qPCR analysis of SPRY1 expression in sputum samples from asthma patients and healthy controls (n = 10). **(B)** Correlation analysis of the relationship between miR-18a-5p and SPRY1 expression levels. **(C)** RT-qPCR analysis of SPRY1 mRNA expression in ASMCs under different treatment conditions (n = 3). **(D)** Western blot analysis of SPRY1 protein expression in ASMCs following different interventions (n = 3). **(E)** Bioinformatic prediction of the putative binding site between miR-18a-5p and the SPRY1 3′UTR. **(F)** Dual-luciferase reporter assay confirming the direct interaction between miR-18a-5p and SPRY1 (n = 3). Data are presented as mean ± SD. **p < 0.01, ***p < 0.001 vs. healthy, Control or WT + NC-mimics group; ##p < 0.01 vs. TGF-β1+in-NC group; &&p < 0.01 vs. TGF-β1+mi-NC group.

RT-qPCR analysis revealed that SPRY1 expression was significantly lower in sputum samples from asthma patients compared with healthy controls (Figure 5A). Furthermore, correlation analysis demonstrated a significant inverse relationship between miR-18a-5p and SPRY1 expression levels across the entire study cohort (Figure 5B), suggesting a regulatory association between the two.

To further validate the regulatory effect of miR-18a-5p on SPRY1 at the cellular level, we assessed SPRY1 mRNA and protein expression in ASMCs. Both RT-qPCR and Western blot analysis showed that TGF-β1 treatment markedly downregulated SPRY1 expression compared with the Control group. Inhibition of miR-18a-5p (TGF-β1+in-miR group) significantly increased SPRY1 mRNA and protein levels, while miR-18a-5p overexpression (TGF-β1+mi-miR group) further reduced SPRY1 expression. No significant differences were observed among the TGF-β1, TGF-β1+in-NC, and TGF-β1+mi-NC groups (Figures 5C,D).

To confirm the direct interaction between miR-18a-5p and SPRY1, bioinformatics analysis predicted a specific binding site for miR-18a-5p in the 3′UTR of SPRY1 mRNA (Figure 5E). A wild-type SPRY1 3′UTR reporter construct (SPRY1-WT) and a corresponding mutant construct (SPRY1-MUT) were generated and co-transfected with miR-18a-5p mimics into HEK293T cells. The dual-luciferase reporter assay revealed that miR-18a-5p mimics significantly suppressed luciferase activity of the SPRY1-WT construct but had no effect on the SPRY1-MUT construct (Figure 5F), confirming the direct binding relationship.

Collectively, these results demonstrate that SPRY1 is a direct target of miR-18a-5p and is negatively regulated by miR-18a-5p at both transcriptional and translational levels.

miR-18a-5p promotes TGF-β1-induced phenotypic transformation of ASMCs by targeting SPRY1 and activating the RAS-MAPK pathway.

**FIGURE 6:**
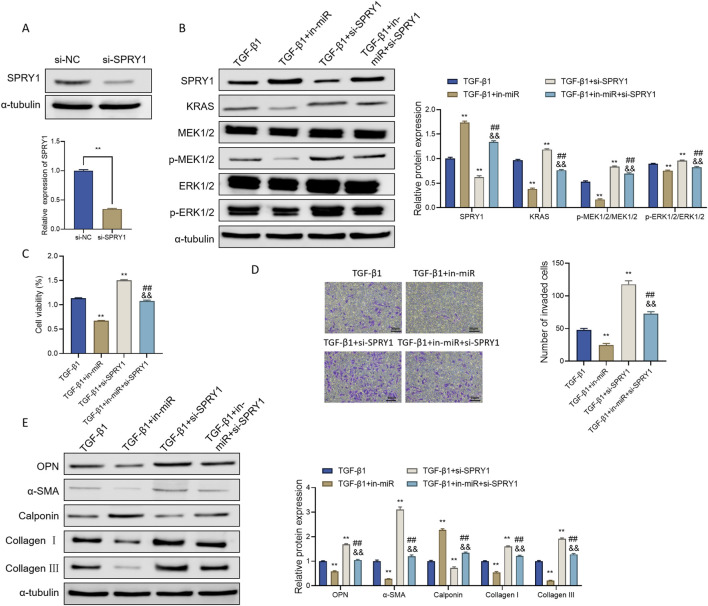
miR-18a-5p promotes TGF-β1-induced phenotypic transformation of ASMCs by targeting SPRY1 and activating the RAS-MAPK signaling pathway. **(A)** Western blot analysis of SPRY1 protein expression in ASMCs under different treatment conditions. **(B)** Western blot analysis of KRAS, MEK1/2, p-MEK1/2, ERK1/2, and p-ERK1/2 in ASMCs following different interventions. **(C)** CCK-8 assay evaluating ASMC viability under various treatment conditions. **(D)** Transwell assay assessing the migration capacity of ASMCs after different treatments. **(E)** Western blot analysis of OPN, α-SMA, Calponin, Collagen I, and Collagen III protein levels in ASMCs under different conditions. Data are presented as mean ± SD (n = 3). **p < 0.01 vs. si-NC or TGF-β1 group; ##p < 0.01 vs. TGF-β1+in-miR group; &&p < 0.01 vs. TGF-β1+si-SPRY1 group.

To confirm that miR-18a-5p modulates ASMC behavior through direct regulation of SPRY1, we first evaluated the knockdown efficiency of SPRY1 using Western blot analysis. Compared with the si-NC group, SPRY1 protein expression was significantly reduced in the si-SPRY1 group (Figure 6A), indicating successful silencing of SPRY1 and providing a basis for subsequent mechanistic studies.

Assessment of RAS-MAPK signaling activity revealed that, relative to the TGF-β1 group, the TGF-β1+in-miR group exhibited markedly decreased expression levels of KRAS, phosphorylated MEK1/2 (p-MEK1/2), and phosphorylated ERK1/2 (p-ERK1/2), along with a downward trend in total MEK1/2 and ERK1/2 levels. Notably, the p-MEK1/2/MEK1/2 and p-ERK1/2/ERK1/2 ratios were also significantly reduced. In contrast, these parameters were substantially elevated in the TGF-β1+si-SPRY1 group.

Further comparison showed that simultaneous suppression of miR-18a-5p and knockdown of SPRY1 led to increased expression of RAS-MAPK pathway proteins compared to the TGF-β1+in-miR group, but to a lesser extent than in the TGF-β1+si-SPRY1 group (Figure 6B). These findings suggest that SPRY1 knockdown activates the RAS-MAPK pathway, while miR-18a-5p inhibition can partially offset this effect.

Functionally, CCK-8 assays revealed that ASMC proliferation was significantly enhanced in the TGF-β1+si-SPRY1 group compared to the TGF-β1 group, whereas cell viability was reduced in the TGF-β1+in-miR group. Co-inhibition of miR-18a-5p and SPRY1 resulted in higher cell viability than in the TGF-β1+in-miR group, but lower than in the TGF-β1+si-SPRY1 group (Figure 6C). Similarly, Transwell assays showed that ASMC migration increased upon SPRY1 knockdown and decreased following miR-18a-5p inhibition. Co-treatment partially restored the migratory capacity compared to TGF-β1+in-miR but remained lower than TGF-β1+si-SPRY1 (Figure 6D). These results indicate that SPRY1 depletion promotes ASMC proliferation and migration, which can be partially reversed by miR-18a-5p downregulation.

At the protein level, Western blot analysis demonstrated that SPRY1 knockdown significantly upregulated the expression of structural proteins associated with the synthetic phenotype, including OPN, α-SMA, Collagen I, and Collagen III, while downregulating the contractile marker Calponin. In the TGF-β1+in-miR + si-SPRY1 group, expression levels of OPN, α-SMA, Collagen I, and Collagen III were higher than those in the TGF-β1+in-miR group, but lower than in the TGF-β1+si-SPRY1 group, with Calponin showing an opposite trend (Figure 6E).

Collectively, these results indicate that SPRY1 deficiency promotes ASMC phenotypic transformation toward a synthetic phenotype, and that miR-18a-5p inhibition can partially reverse this transition. Therefore, miR-18a-5p promotes TGF-β1-induced phenotypic switching of ASMCs by downregulating SPRY1, thereby relieving its inhibitory effect on the RAS-MAPK signaling pathway.

## Discussion

To the best of our knowledge, this study is the first to systematically explore the potential role and underlying mechanisms of miR-18a-5p in airway remodeling in asthma. Analysis of clinical samples revealed a significant upregulation of miR-18a-5p in patients with asthma. In an *in vitro* TGF-β1-induced ASMC model, we found that miR-18a-5p overexpression markedly promoted ASMC proliferation and migration, and upregulated the expression of structural proteins including α-SMA, OPN, Collagen I, and Collagen III, suggesting that miR-18a-5p facilitates the phenotypic switch of ASMCs toward a synthetic phenotype. Mechanistically, miR-18a-5p promotes this transition by targeting SPRY1 and relieving its inhibitory effect on the RAS-MAPK signaling pathway, thereby synergizing with TGF-β1 to induce phenotypic transformation. These findings provide new insights into the mechanisms of airway remodeling in asthma and identify a novel therapeutic target.

A key challenge in studying airway remodeling *in vitro* lies in accurately replicating the pathological features observed in asthma patients. In this study, we employed TGF-β1 to induce asthma-like phenotypic changes in ASMCs. TGF-β1 has been shown to be highly expressed in the airway tissues of asthma patients (Ojiaku et al., 2017), and extensive experimental evidence demonstrates that it can stimulate the proliferation, migration, and differentiation of various bronchial cells, as well as promote extracellular matrix and fibrotic component synthesis—leading to airway wall thickening and narrowing (Aghasafari et al., 2019). Our findings showed that TGF-β1 treatment significantly enhanced ASMC proliferation and migration and increased the expression of synthetic phenotype-related structural proteins. These results are consistent with *in vitro* asthma models reported by Li et al. and Alvarez-Santos et al. (Alvarez-Santos et al., 2022; Li et al., 2020), confirming that TGF-β1 stimulation successfully mimicked the phenotypic remodeling observed in asthmatic ASMCs.

We further demonstrated the regulatory role of miR-18a-5p in ASMC phenotypic transformation at both the cellular behavior and molecular marker levels. Functionally, overexpression of miR-18a-5p enhanced ASMC proliferation and migration, while its inhibition suppressed these capabilities. It is well established that the transition of ASMCs from a contractile (quiescent) phenotype to a synthetic phenotype is characterized by increased functional activity, including uncontrolled proliferation and migration, which contribute to airway wall thickening and remodeling (Li et al., 2022; Halayko et al., 2006). At the molecular level, miR-18a-5p overexpression significantly upregulated OPN, α-SMA, Collagen I, and Collagen III, and downregulated Calponin; miR-18a-5p inhibition produced opposite effects. Among these, α-SMA is a marker of contractile function whose overexpression contributes to airway constriction in early asthma; OPN is involved in inflammatory cell recruitment; Collagen I and III are major extracellular matrix components whose elevation indicates increased matrix deposition; and Calponin is a classical marker of the contractile phenotype, with its reduction indicating phenotypic switching (Sharma et al., 2014; Kaczmarek et al., 2019; Liu et al., 2024). In addition to these phenotypic markers, we observed that miR-18a-5p modulated the expression of migration-associated proteins, including Integrin β1, FAK/Paxillin signaling components, and MMP9, under TGF-β1 stimulation. These molecules are critically involved in cell–matrix adhesion, cytoskeletal remodeling, and extracellular matrix degradation, providing further mechanistic support for the pro-migratory effect of miR-18a-5p in ASMCs. Collectively, our results support the critical role of miR-18a-5p in promoting ASMC phenotypic transformation through both functional and structural changes, which represents a novel finding of this study.

After confirming the phenotypic regulatory function of miR-18a-5p, we further investigated its molecular mechanism and focused on the classical RAS-MAPK pathway. This pathway, initiated by RAS and involving sequential activation of MEK1/2 and ERK1/2, is highly conserved and regulates key biological processes such as cell proliferation, differentiation, migration, and survival. Its dysregulation has been implicated in inflammatory, autoimmune, and neoplastic diseases (Trujillo, 2011). In asthma, abnormal RAS-MAPK activation contributes to airway wall structural and functional remodeling (Freund-Michel and Frossard, 2008). In our study, overexpression of miR-18a-5p elevated KRAS, p-MEK1/2, and p-ERK1/2 levels, indicating its positive regulation of the RAS-MAPK pathway, which likely mediates its pro-remodeling effects.

As a class of non-coding small RNAs, miRNAs exert biological functions by base-pairing with the 3′UTR of target mRNAs, leading to their degradation or translational repression (Ho et al., 2022). Identifying the direct target gene by which miR-18a-5p activates the RAS-MAPK pathway is therefore critical. Based on bioinformatic predictions and literature review, we focused on SPRY1. As a member of the Sprouty protein family, SPRY1 can translocate to the cell membrane upon activation and, through competitive binding with Grb2, block RAS activation and downstream MEK/ERK signaling (Liu et al., 2014; Hanafusa et al., 2002). Our dual-luciferase reporter assay provided functional evidence supporting a regulatory interaction between miR-18a-5p and the SPRY1 3′UTR. Moreover, knockdown of SPRY1 reversed the inhibitory effects of miR-18a-5p inhibition on ASMC phenotype and behavior, confirming that miR-18a-5p promotes phenotypic switching primarily by downregulating SPRY1 and thereby lifting its suppression of the RAS-MAPK pathway.

It should be noted that although miR-18a-5p expression was examined in human sputum samples, mechanistic experiments were conducted using mouse-derived ASMCs to enable controlled and reproducible *in vitro* analyses, which may introduce translational limitations. Bioinformatic analyses using TargetScan and ENCORI (starBase) did not identify a canonical or cross-species conserved 3′untranslated region (3′UTR) seed match between miR-18a-5p and SPRY1. However, the absence of predicted canonical binding sites does not exclude potential miRNA-mediated regulation, as non-canonical interactions, transcript-specific 3′UTR variants, or non-3′UTR binding may not be captured by current algorithms. In this study, the miR-18a-5p–SPRY1 regulatory relationship was supported by functional evidence from dual-luciferase reporter and loss-of-function assays rather than sequence conservation alone, and further validation in human ASMCs and *in vivo* models is warranted.

Nevertheless, this study has some limitations. First, the experimental model was restricted to *in vitro* TGF-β1-stimulated ASMCs, which only partially recapitulate the *in vivo* airway remodeling in asthma. Future *in vivo* studies, including animal models, are needed to validate the role of miR-18a-5p in a physiological context. Second, miRNAs are known for their multi-targeting properties and may regulate multiple downstream mRNAs and signaling pathways, and the expression of any single target gene can also be influenced by multiple upstream factors. As a result, the correlation between SPRY1 and miR-18a-5p in sputum samples appeared modest, which may also reflect the limited sample size. Nevertheless, *in vitro* RT-qPCR, Western blot, and dual-luciferase assays consistently demonstrated that SPRY1 is a direct functional target of miR-18a-5p, supporting the robustness of our mechanistic conclusions. High-throughput transcriptomic and proteomic analyses are warranted to elucidate the broader regulatory network and biological functions of miR-18a-5p. Another limitation of this study is the relatively small sample size of clinical sputum specimens, which may limit the statistical power and generalizability of the findings. Therefore, the observed associations between miR-18a-5p expression and asthma should be interpreted as exploratory, and validation in larger, independent cohorts is warranted.

## Conclusion

To sum up, this study demonstrated that miR-18a-5p is upregulated in the sputum of patients with asthma. *In vitro*, miR-18a-5p was shown to promote proliferation, migration, and structural protein expression in TGF-β1-induced ASMCs, thereby facilitating their phenotypic transformation from a contractile to a synthetic state. Mechanistically, miR-18a-5p exerts these effects primarily by targeting and suppressing SPRY1, thereby relieving its inhibitory influence on the RAS-MAPK signaling pathway. These findings provide novel insights into the pathogenic role of miR-18a-5p in asthma-related airway remodeling and offer a theoretical foundation for the development of miRNA-based therapeutic strategies for asthma.

## Data Availability

The original contributions presented in the study are included in the article/supplementary material, further inquiries can be directed to the corresponding author.
